# Lysergic acid diethylamide (LSD) promotes social behaviour through 5-HT_2A_ and ampa in the medial prefrontal cortex (MPFC)

**DOI:** 10.1192/j.eurpsy.2021.1112

**Published:** 2021-08-13

**Authors:** A. Markopoulos, A. Inserra, D. De Gregorio, G. Gobbi

**Affiliations:** Psychiatry, McGill University, Montreal, Canada

**Keywords:** LSD, sociability, electrophysiology, behavior

## Abstract

**Introduction:**

Autism Spectrum Disorder and Social Anxiety Disorder are mental illnesses characterized by a dysfunction in social behavior (SB); a phenomenon largely mediated by the medial prefrontal cortex (mPFC). Clinical studies have demonstrated that lysergic acid diethylamide (LSD), a partial agonist of the 5-HT_2A_ receptor, can promote SB. However, its mechanism of action on SB is unknown.

**Objectives:**

To assess the effects of repeated LSD administration on social behavior in mice and to identify which mPFC receptors mediate LSD’s behavioral effects.

**Methods:**

Eight-week-old C57BL/6J male mice received vehicle or repeated LSD (30 μg/kg/day i.p. for 7 days) as well the selective 5-HT_2A_ receptor antagonist MDL, or the AMPA receptor antagonist NBQX. Twenty-four hours following the last injection, mice underwent the Direct Social Interaction Test and the Three-Chamber Test (TCT) to assess sociability and preference for social novelty. in vivo electrophysiological recordings were performed in mice treated with vehicle or LSD using multi-barrelled electrodes for microiontophoretic ejections of the selective 5-HT_2A_ receptor agonist DOI or the selective AMPA receptor agonist quisqualate on mPFC pyramidal neurons.

**Results:**

Repeated treatment with low doses of LSD increased the interaction time in the DSI as well as sociability and social novelty indices in the TCT. These pro-social effects were blocked by the intra-PFC administration of both 5-HT_2A_ and AMPA antagonists. LSD also potentiated, in a current-dependent manner, the excitatory response of mPFC neurons to 5-HT_2A_and AMPA agonists.

**Conclusions:**

Repeated, low doses of LSD increases social behavior via a mechanism of action that is mediated by 5-HT_2A_ and AMPA in the mPFC.

